# Association of MDM2 SNP309 Variation with Lung Cancer Risk: Evidence from 7196 Cases and 8456 Controls

**DOI:** 10.1371/journal.pone.0041546

**Published:** 2012-07-23

**Authors:** Wenlei Zhuo, Liang Zhang, Bo Zhu, Junjun Ling, Zhengtang Chen

**Affiliations:** 1 Institute of Cancer, Xinqiao Hospital,Third Military Medical University, Chongqing, China; 2 Department of Environmental Hygiene, College of Preventive Medicine, Third Military Medical University, Chongqing, China; 3 Department of Otolaryngology, Southwest Hospital, Third Military Medical University, Chongqing, China; Ohio State University Medical Center, United States of America

## Abstract

**Background:**

Evidence suggests that MDM2 T309G polymorphism may be a risk factor for several cancers. Increasing investigations have been conducted on the association of MDM2 T309G polymorphisms with lung cancer risk and have yielded conflicting results. Previous meta-analyses on this issue have reported inconclusive data. The aim of the present study was to derive a more precise estimation of the relationship.

**Methods and Findings:**

Updated meta-analyses examining the association between MDM2 T309G polymorphism and lung cancer risk were performed. Separate analyses on ethnicity, smoking status, histological types and gender as well as source of controls were also implemented. Eligible studies were identified for the period up to Feb 2012. Lastly, ten publications including eleven case-control studies were selected for analysis. The overall data failed to indicate a significant association between MDM2 T309G polymorphism and lung cancer risk (GG vs TT OR = 1.14; 95%CI = 0.95−1.37; dominant model: OR = 1.05; 95%CI = 0.92−1.19; recessive model: OR = 1.12; 95%CI = 0.99−1.27). In a subgroup analysis by smoking status, increased lung cancer risk was shown among never-smokers (GG vs TT: OR = 1.76; 95%CI = 1.36−2.29; dominant model: OR = 1.48; 95%CI = 1.22−1.81; recessive model: OR = 1.37; 95%CI = 1.11−1.69). In subgroup analysis by gender, elevated risk was presented among women under a recessive model (OR = 1.29; 95%CI = 1.04−1.59). In the subgroup analysis by ethnicity, histological types and source of controls, no marked associations were observed.

**Conclusions:**

Compared to the previous meta-analyses, the results of this study confirmed that MDM2 T309G polymorphism might be a risk factor for lung cancer among never-smokers. However, the data failed to suggest a marked association between the G allele of MDM2 T309G and lung cancer risk among Asians. More interestingly, subgroup analysis by gender indicated that homozygous GG alleles might raise lung cancer risk among females.

## Introduction

Lung cancer is the most commonly diagnosed cancer as well as the leading cause of cancer death worldwide [1]. Evidence suggests that cigarette smoking is its established major risk factor [2]. In addition, exposure to several environmental chemical carcinogens such as airborne genotoxic carcinogens and arsenic are also considered risk factors [3]. The mechanism of lung tumorigenesis is not fully understood. Interestingly, lung cancer develops only in a small proportion of people exposed to environmental risk factors and extensive tobacco consumption, implying that genetic factors might play a critical role in its carcinogenic mechanisms. Epidemiological evidence suggests that complex interactions between many genetic and environmental factors are important in carcinogenesis of lung carcinoma [4].

Genetic factors involved in lung cancers have been extensively studied and to date several genetic polymorphisms have been identified as candidates by meta-analyses. Previous studies indicated that variations of some genes such as CYP1A1 [5], GSTM1 [6], CYP1B1 [7] and TP53 [8] may be associated with increased susceptibility to lung cancer. Conversely, polymorphisms of NAT2 [9], ERCC1 [10] and TNF alpha [11] might not have significant association with tumorigenesis of lung cancer. However, although these genetic factors are important, only a few gene polymorphisms associated with lung cancer susceptibility have been identified.

Murine double minute-2 (MDM2) is a key negative regulator of the P53 tumor suppressor pathway that has been suggested to be mutated in a variety of cancers [12]. MDM2 can bind directly to the P53 protein and inhibit its activity, thus resulting in its degradation via the ubiquitination pathway [13]. Over-expression of MDM2 has been detected in some malignancies; therefore, MDM2 targeting via utilization of antagonists has been indicated as a potential approach to anti-cancer therapy [14].

**Figure 1:**
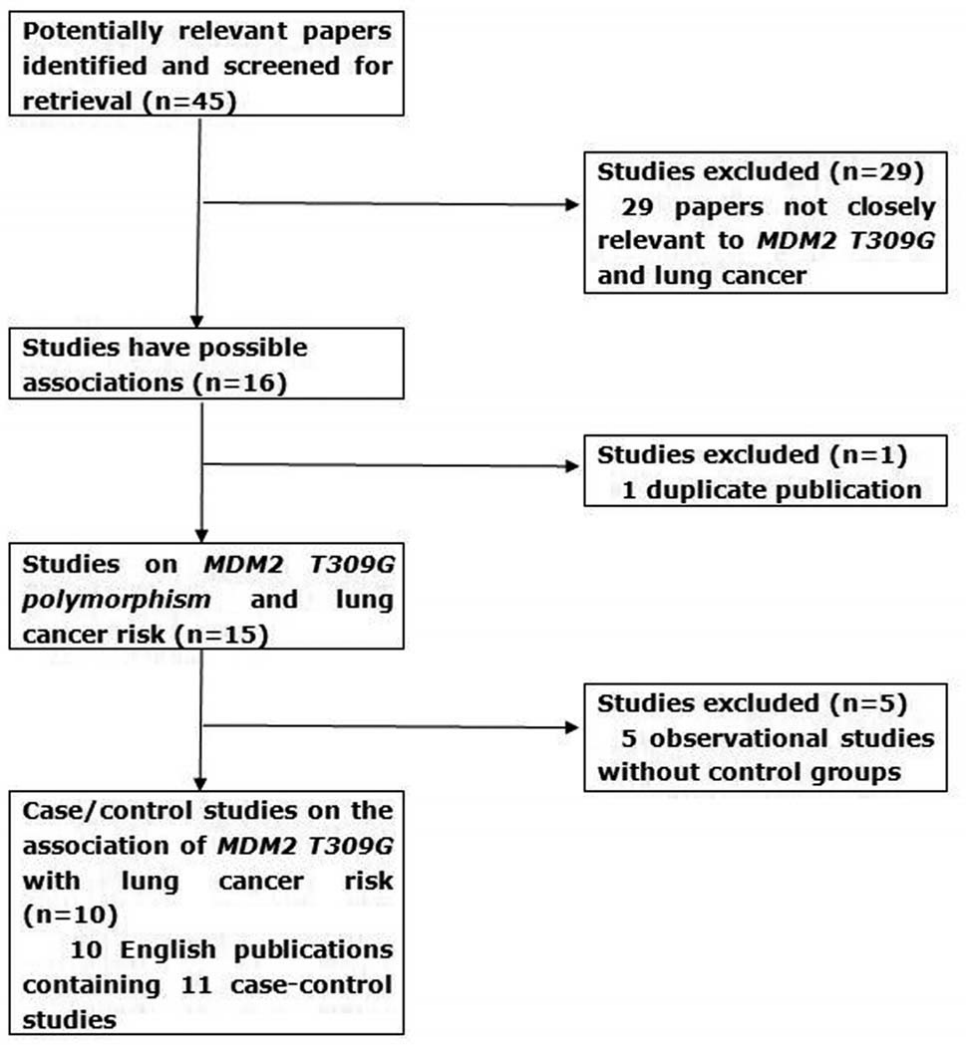
The flow diagram of included/excluded studies.

A MDM2 single nucleotide polymorphism at the 309^th^ nucleotide in the first intron (rs2279744), with a T to G change, could increase the affinity for stimulatory protein (Sp) 1 binding and result in increased MDM2 expression and subsequent attenuation of the P53 pathway [15]. This polymorphism has been associated with several cancers including lung cancer. Published data on the possible association of MDM2 T309G polymorphism with lung cancer have generated inconclusive results.

Previously, Gui et al. and Bai et al. conducted meta-analyses concerning the relationship between MDM2 T309G polymorphism and lung cancer risk [16], [17]. These reports indicated a possible association of the MDM2 309G allele as a low-penetrant risk factor for lung cancer in Asians. However, the two meta-analyses only contained published data from prior to 2008. In addition, only ethnicity and smoking status were considered for the subgroup analysis.

In the present study, we carefully conducted a search and retrieved the possible publications up to Feb 2012. Then, we performed an updated meta-analysis that increases statistical power to derive a more precise estimation of the relationship.

**Table 1:** General characteristics of the studies included in the present meta-analysis.

First Author	Publication Year	Number of Cases(male/female)	Number of Controls(male/female)	Histological typesof cases	Type of controls	Mean Age, year(Cases/Controls)	Racial decent	Country
Hu	2006	717 (527/190)	1083 (787/296)	245 SQCC; 278 AC; 48 SCLC; 146 others	Cancer-free controls (age-, sex-, residential area-matched; HB)	NA/NA	Asian	China
Li	2006	1026 (542/484)	1145 (558/587)	224 SQCC; 503 AC; 200 NSCLC; 71 SCLC; 28 others	healthy controls (sex-,ethnicity-,age-,smoking status-matched; HB)	NA/NA	Caucasian	USA
Lind	2006	341 (258/83)	412 (315/97)	341 NSCLC	healthy controls (PB)	63.3/63.5	Caucasian	Norway
Park	2006	582 (467/115)	582 (467/115)	270 SQCC; 205 AC; 10 LCC; 97 SCLC	healthy volunteers (age-, sex-matched; HB)	61.3/60.2	Asian	Korea
Pine	2006	504 (NA/NA)	680 (NA/NA)	504 NSCLC	healthy controls (gender-,ethnicity-,age-,smoking history-matched; HB)	NA/NA	African and Caucasian	USA
Zhang	2006	1106 (805/301)	1420 (1029/391)	476 SQCC; 361 AC; 269 others	Cancer-free controls (age-, sex-matched; PB)	NA/NA	Asian	China
Liu	2008	1787 (921/866)	1360 (605/755)	423 SQCC; 1055 AC; 154 LCC; 155 others	healthy controls (HB)	67/60	Caucasian	Canada
Mittelstrass	2008	635 (406/229)	1300 (819/481)	222 AC; 218 other NSCLC; 152 SCLC; 42 others	healthy controls (gender-, age-, matched; PB)	45.2/45.0	Caucasian	Germany
Chua	2010	123 (NA/NA)	159 (NA/NA)	14 SQCC; 85 19 others	healthy controls (age-, matched; HB)	62.0/63.4	Asian	Singapore
Kohno	2011	377 (340/37)	325 (185/140)	377 SQCC	non-cancerous controls (HB)	62.7/62.5	Asian	Japan

NA: not available; AC: adenocarcinoma; LCC: large cell carcinoma; SQCC: squamous cell carcinoma; SCLC: small cell lung carcinoma; NSCLC: non-small cell lung carcinoma; PB: population-based; HB: hospital-based.

## Materials and Methods

### 1. Literature Search Strategy

We carried out a search in the Medline, EMBASE, OVID, Sciencedirect, and Chinese National Knowledge Infrastructure (CNKI) without a language limitation, covering all papers published up to Feb 2012, with a combination of the following keywords: *Murine double minute-2, MDM2, lung, neoplasm, tumor, cancer, variation* and *polymorphism*. All searched studies were retrieved and the bibliographies were checked for other relevant publications. Review articles and bibliographies of other relevant studies identified were hand searched to find additional eligible studies.

**Table 2:** Distribution of MDM2 T309G genotypes among lung cancer cases and controls included in the present meta-analysis.

First Author	Genotypingmethod	Cases			Controls			HWE (control)	
		GG	GT	TT	GG	GT	TT	Chi-squre	P
Hu	PIRA-PCR	178	373	166	271	538	274	0.045	.>0.05
Li	PIRA-PCR	135	472	419	164	573	408	2.692	>0.05
Lind	Taqman	55	156	130	44	207	161	3.563	>0.05
Park	PCR-RFLP	189	280	113	161	299	122	0.601	>0.05
Pine(African)	MGB Eclipse	2	20	111	5	47	203	1.310	>0.05
Pine(Caucasian)	MGB Eclipse	54	167	150	52	187	182	0.136	>0.05
Zhang	ARMS-PCR	296	561	249	291	711	418	0.128	>0.05
Liu	TaqMan	283	802	702	199	631	530	0.253	>0.05
Mittelstrass	MALDITOF	70	293	270	149	598	547	0.562	>0.05
Chua	Sequencing	29	65	29	51	83	25	0.841	>0.05
Kohno	Pyrosequencing	126	183	68	95	151	79	1.525	>0.05

### 2. Inclusion Criteria

The following criteria were used for the literature selection: first, studies should concern association of MDM2 T309G polymorphism with lung cancer risk; second, studies must be observational studies (Case–control or cohort); and third, papers must offer the size of the sample, odds ratios (ORs) and their 95% confidence intervals (CIs), the genetic distribution or the information that can help infer the results. After rigorous searching, we reviewed all papers in accordance with the criteria defined above for further analysis.

**Table 3:** Main results of the pooled data in the present meta-analysis.

	No. (cases/controls)	GG vs TT			(GG+GT) vs TT			GG vs (GT+TT)		
		OR (95%CI)	P	P (Q-test)	OR (95%CI)	P	P (Q-test)	OR (95%CI)	P	P (Q-test)
Total	7196/8456	1.14 (0.95–1.37)	0.164	0.001	1.05 (0.92–1.19)	0.502	0.001	1.12 (0.99–1.27)	0.084	0.035
Ethnicity										
Asian	2905/3569	1.23 (0.91–1.66)	0.181	0.004	1.18 (0.96–1.46)	0.108	0.036	1.14 (0.93–1.40)	0.206	0.024
Caucasian	4158/4632	1.05 (0.86–1.27)	0.654	0.106	0.96 (0.86–1.07)	0.434	0.218	1.08 (0.92–1.27)	0.368	0.183
African	133/255	0.73 (0.14–3.83)	0.711	–	0.77 (0.45–1.34)	0.360	–	0.76 (0.15–3.99)	0.749	–
Smoking status										
Ever smoking	3309/3056	1.25 (0.78–1.99)	0.354	0.000	1.08 (0.77–1.51)	0.652	0.000	1.21 (0.90–1.62)	0.205	0.004
Never smoking	815/1481	1.76 (1.36–2.29)	0.000	0.926	1.48 (1.22–1.81)	0.000	0.501	1.37 (1.11–1.69)	0.004	0.994
Histological types										
AC	2355/5802	1.24 (0.90–1.70)	0.188	0.003	1.07 (0.88–1.31)	0.503	0.016	1.21 (0.96–1.52)	0.102	0.023
SQCC	1770/4833	1.12 (0.83–1.52)	0.456	0.012	1.03 (0.80–1.34)	0.814	0.003	1.13 (0.96–1.33)	0.150	0.242
Source of control										
HB	5116/5330	1.06 (0.88–1.27)	0.549	0.053	1.00 (0.87–1.16)	0.993	0.026	1.06 (0.94–1.18)	0.336	0.310
PB	2080/3126	1.36 (0.92–2.01)	0.121	0.013	1.15 (0.88–1.49)	0.301	0.014	1.28 (0.96–1.71)	0.090	0.055
Gender										
Male	2253/2760	0.99 (0.75–1.31)	0.951	0.064	0.94 (0.79–1.13)	0.533	0.091	1.02 (0.81–1.29)	0.843	0.098
Female	1740/2033	1.28 (0.95–1.73)	0.098	0.165	1.01 (0.86–1.18)	0.926	0.313	1.29 (1.04–1.59)	0.019	0.336

AC: adenocarcinoma; SQCC: squamous cell carcinoma; PB: population-based; HB: hospital-based.

### 3. Data Extraction

Data were carefully extracted from all eligible publications independently by two of the authors **(Zhuo and Zhang)** according to the inclusion criteria mentioned above. For conflicting evaluations, an agreement was reached following a discussion. If a consensus could not be reached, another author was consulted to resolve the dispute and then a final decision was made by the majority of the votes. Extracted information was entered into a database.

### 4. Statistical Analysis

The odds ratio (OR) of MDM2 T309G polymorphisms and lung cancer risk was estimated for each study. The pooled ORs were performed for additive model (GG versus TT), dominant model (GG+GT versus TT) and recessive model (GG versus GT+TT). For detection of any possible sample size bias, the OR and its 95% confidence interval (CI) for each study was plotted against the number of participants. *I^2^* metric was applied to quantify heterogeneity between the included studies (*I^2^* = 0–25%, no heterogeneity; *I^2^* = 25–50%, moderate heterogeneity; *I^2^*>50%, large heterogeneity) [18]. Moreover, a chi-square based Q statistic test was performed to assess heterogeneity. If *P*>0.1 for a given Q-test indicated a lack of heterogeneity among the studies, then ORs were pooled according to the fixed-effect model (Mantel-Haenszel) [19]. Otherwise, the random-effect model (DerSimonian and laird) was used [20]. The significance of the pooled ORs was determined using the Z-test. The Hardy-Weinberg equilibrium (HWE) was assessed via Fisher’s exact test.

Publication bias was assessed by visual inspection of funnel plots [21], in which the standard error of log (OR) of each study was plotted against its log (OR). An asymmetric plot indicates a possible publication bias. Symmetry of the funnel plot was further evaluated by Egger’s linear regression test [22]. Statistical analysis was undertaken using the program Review Manager 5 and STATA 11.0 softwares (Stata Corporation, Texas).

## Results

### 1. Study Characteristics

Possible relevant publications were retrieved and screened. As shown in **Figure 1**, sixteen publications were preliminary eligible, of these one was excluded because of being a duplicate publication [23] whose relevant data had been published in one included study [24]. Next, five papers were discarded because they were not case-control studies [25], [26], [27], [28], [29]. Consequently, ten publications containing eleven case-control studies regarding MDM2 T309G polymorphism with respect to lung carcinoma were included [24], [30], [31], [32], [33], [34], [35], [36], [37], [38].

**Figure 2:**
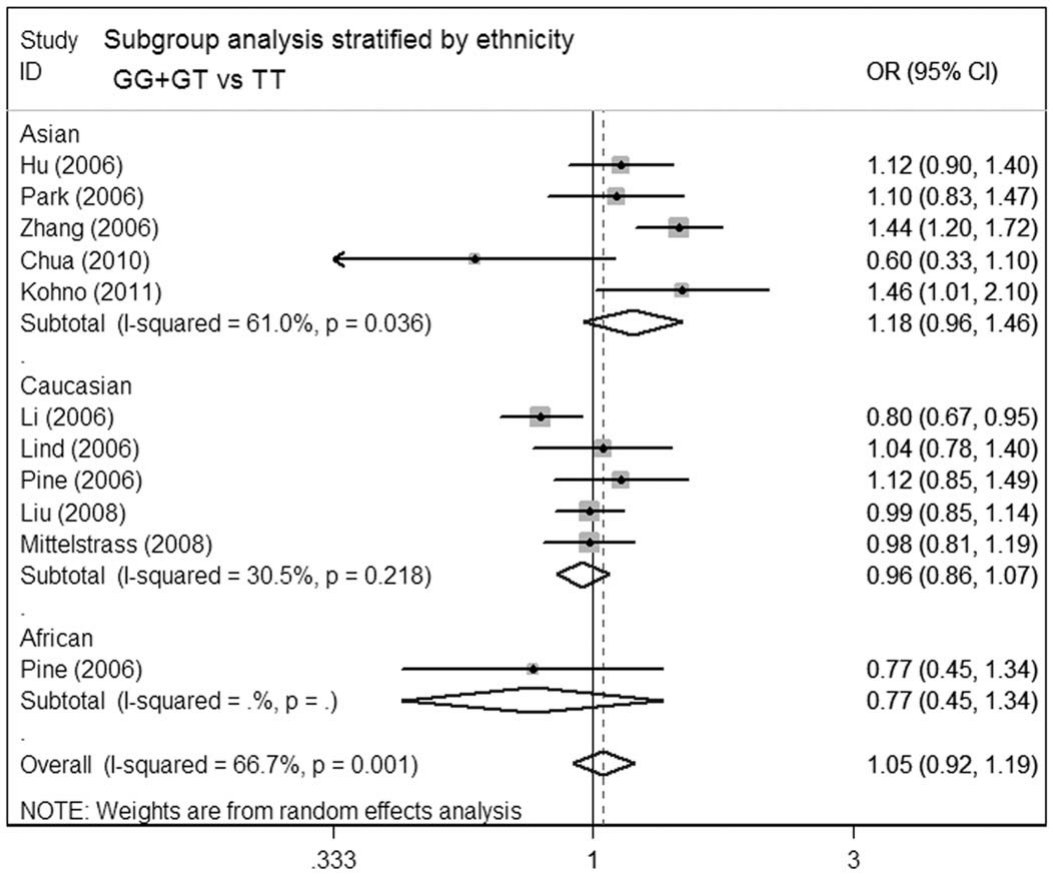
Meta-analysis for the association of lung cancer risk with MDM2 T309G polymorphism (GG+GT versus TT; stratified by ethnicity).

**Figure 3:**
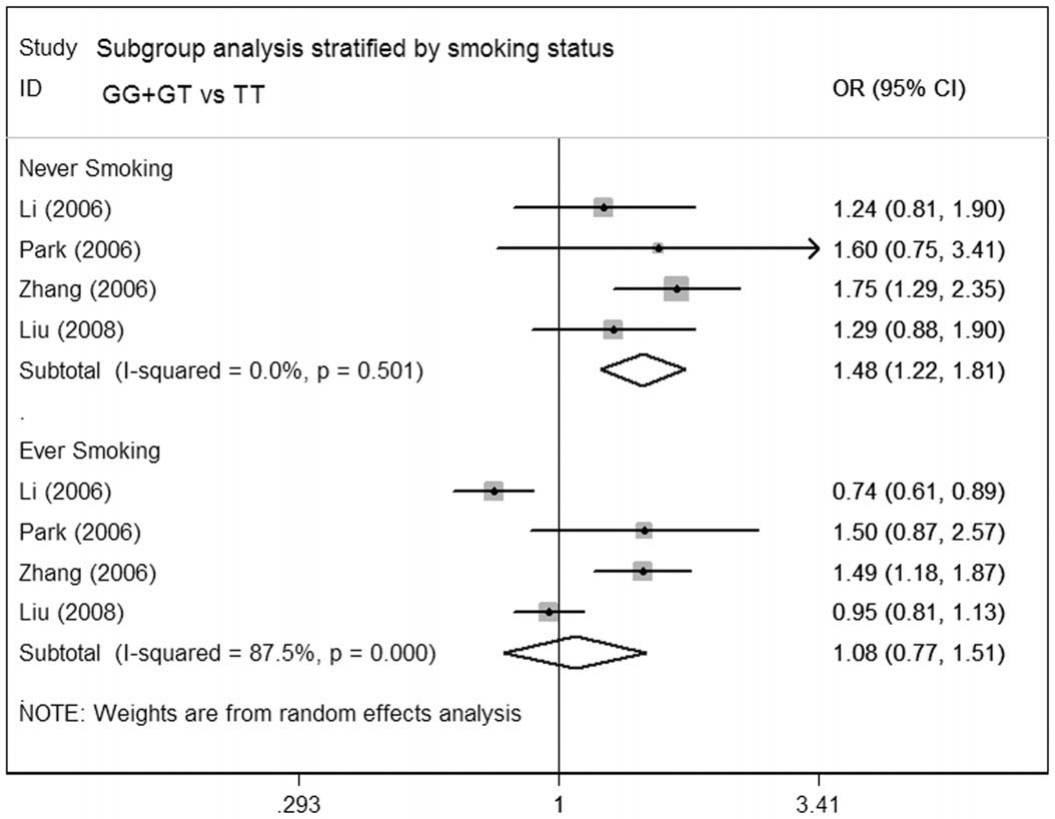
Meta-analysis for the association of lung cancer risk with MDM2 T309G polymorphism (GG+GT versus TT; stratified by smoking status).

**Figure 4:**
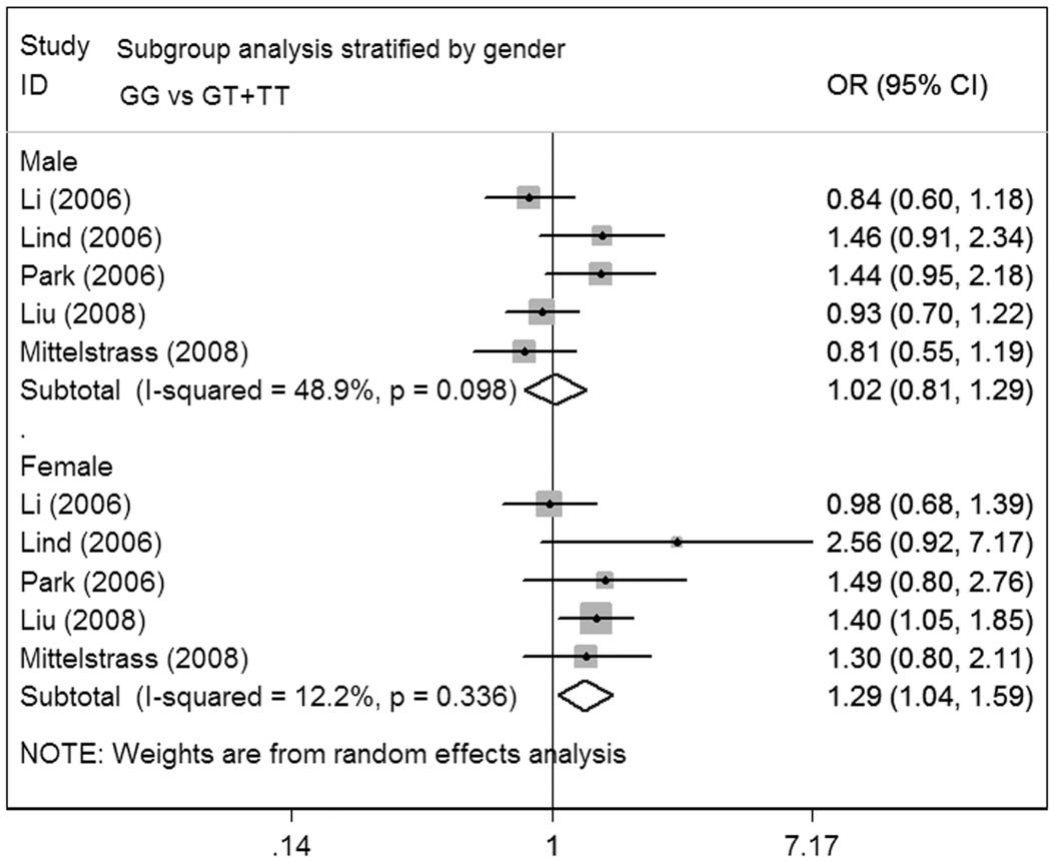
Meta-analysis for the association of lung cancer risk with MDM2 T309G polymorphism (GG versus GT+TT; stratified by gender).

Of the selected publications, all were written in English. We established a database according to the extracted information from each study. The relevant information was listed in **Table 1**. According to this table, the first author and the number and characteristics of cases and controls for each study as well as other necessary information were presented.

In the included studies, there were five groups of Caucasians [33], [34], [35], [36], [37], five of Asians [24], [30], [31], [32], [38] and one of Africans [37]. In the study by Pine et al., cases and controls were separated as Africans and Caucasians according to their race [37]. Therefore, the relevant data were divided for further analysis into two groups according to ethnicity.

Information about histological category could be extracted from six studies [24], [32], [33], [35], [36], [38], of which the study by Kohno et al. [32] provided only data on squamous cell carcinoma whereas the study by Mittelstrass et al. [36] presented data on adenocarcinoma. The remaining four studies provided data on both squamous cell carcinoma and adenocarcinoma. As for smoking status, sufficient data were available in four studies [24], [33], [35], [38]. Moreover, sufficient information regarding gender could be extracted from five studies [24], [33], [34], [35], [36].

The distributions of MDM2 T309G genotype as well as the genotyping methods of the included studies are presented in **Table 2**. The genetic distributions of the control groups in all studies were consistent with HWE.

**Figure 5:**
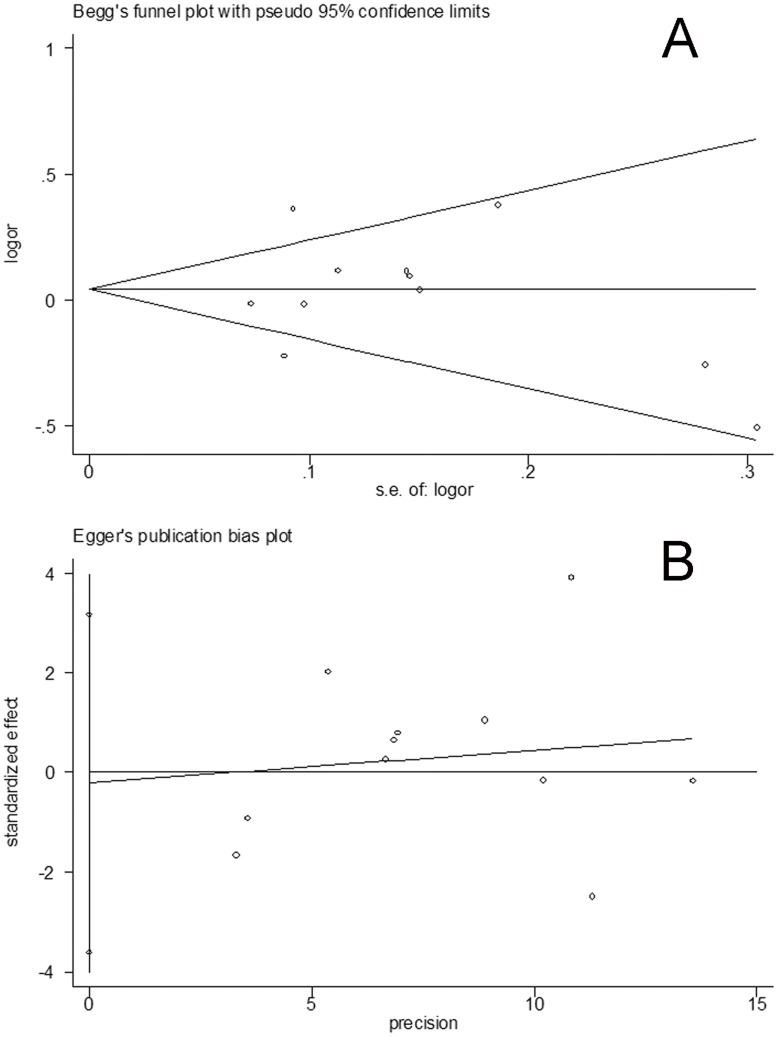
Publication bias test for the overall data (GG+GT versus TT; A: Funnel plot; B: Egger’s linear regression test).

### 2. Test of Heterogeneity

As shown in **Table 3**, we analyzed the heterogeneity for the three genetic models. Evident heterogeneity was observed for overall data in the additive model (*P* = 0.001 for Q-test; *I^2^* = 67.5%), dominant model ((*P* = 0.001 for Q-test; *I^2^* = 66.7%) and recessive model (*P* = 0.035 for Q-test; *I^2^* = 48.7%).

However, when subgroup analyses regarding ethnicity, smoking status and histological types, gender as well as source of controls were further conducted, we found a reduced or loss of heterogeneity in some of the subgroups.

### 3. Meta-analysis Results

**Table 3** lists the main results of the meta-analysis. The overall data in the additive (OR = 1.14; 95%CI = 0.95–1.37; P = 0.001 for heterogeneity), dominant (OR = 1.05; 95%CI = 0.92–1.19; P = 0.001 for heterogeneity) and recessive (OR = 1.12; 95%CI = 0.99–1.27; P = 0.035 for heterogeneity) did not show a marked association of MDM2 T309G polymorphism with lung cancer risk, indicating that individuals with homozygous G allele might not have an increased lung cancer risk compared with those who carry wild-type T allele.

In subgroup analyses **(** **Table 3** **)**, when data were stratified according to ethnicity, no increased lung cancer risk was observed among Asians, Caucasians and Africans **(** **Figure 2** **)**. Likewise, when data were stratified by histological types and source of controls, no significant increased lung cancer risk were observed. Nevertheless, in subgroup analysis regarding smoking status, increased lung carcinoma susceptibility was shown among the never-smoking subgroup (GG vs TT: OR = 1.76; 95%CI = 1.36–2.29; P = 0.926 for heterogeneity; dominant model: OR = 1.48; 95%CI = 1.22–1.81; P = 0.501 for heterogeneity; recessive model: OR = 1.37; 95%CI = 1.11–1.69; P = 0.994 for heterogeneity), suggesting that G allele of MDM2 might increase lung cancer risk among individuals who have no smoking history. No significant association of MDM2 polymorphism with lung cancer risk was shown in the smoking group **(** **Figure 3** **)**. In subgroup analysis by sex, the data indicated no associations regarding lung cancer risk among male subgroup. However, as for the female group, increased lung cancer risk was shown under the recessive model (OR = 1.29; 95%CI = 1.04–1.59; P = 0.336 for heterogeneity) **(** **Figure 4** **)**.

### 4. Sensitivity Analysis

When the effect-models were changed, the significance of the overall data for the three models was not statistically altered (data not shown). Additionally, we also conducted one-way sensitivity analysis [39] to evaluate the stability of the meta-analysis. The statistical significance of the results was not altered when any single study was omitted (data not shown), suggesting stability and credibility of the results.

### 5. Bias Diagnostics

Funnel plots were created for assessment of possible publication biases. Then, Egger’s linear regression tests were used to assess the symmetries of the plots. The funnel plots appeared to be symmetrical for the three models of the overall data **(** **Figure 5A** **)**. Additionally, results of the Egger’s tests also support the notion that the funnel plots were symmetrical (GG vs TT: t = −0.58, P>0.05; dominant model: t = −0.14, P>0.05; recessive model: t = −0.72, P>0.05) **(** **Figure 5B** **)**.

## Discussion

For the overall data, the results showed that MDM2 T309G might not have a correlation with increased lung cancer risk. However, the subgroup analyses revealed an increased lung cancer risk among never-smokers who carry the G allele. Moreover, homozygous GG may increase lung cancer susceptibility among women.

Several genome-wide association studies (GWASs) on SNPs have identified three genomic loci at 15q25, 5p15 and 6p21, which associate with the risk of lung cancer in Caucasians [40], [41], [42]. Then, variations at chromosome 12p13.33 have been found to influence lung cancer risk among Europeans [43]. More recently, other genomic regions such as 18p11.22, 3q28, 13q12.12, and 22q12.2 have been identified to confer lung cancer susceptibility in Asians [44], [45], [46]. Nevertheless, associations of MDM2 polymorphisms were not investigated in the GWASs mentioned above because of the lack of probes to discriminate the polymorphisms used for GWASs [32].

Previous meta-analyses have been conducted on the association of MDM2 T309G polymorphisms with several other cancer risks. Reports suggest that MDM2 T309G variation could increase risk of sarcoma, endometrial, hepatocellular and breast cancers [47], [48], [49], [50]. Conversely, G allele may play a preventive role for head and neck cancer among Caucasians [51]. Thus, the roles of MDM2 T309G polymorphisms might differ in different cancers.

For lung carcinoma, in the studies by Bai et al. and Gui et al. only seven reports with eight groups containing 6063 cases and 6678 controls were involved [16], [17]. In the present meta-analysis, we carefully searched possible publications. Ten publications comprising eleven groups with 7196 cases and 8456 controls were selected. Compared with the mentioned meta-analyses by Bai et al. and Gui et al., one study involving Caucasians [36] and two recent studies involving Asians [30], [32] were added in the present updated meta-analysis. Moreover, in addition to ethnicity and smoking status, subgroup analysis regarding histological types, source of controls and sex were also carried out.

Smoking is an established risk factor for lung cancer and our data suggest that G allele carriers may have an approximately 48 percent higher lung cancer risk than the homozygous TT carriers who have no smoking history. However, the G allele seems to exert little effect on smokers. In the previous meta-analyses by Bai et al. and Gui et al., only the study by Bai et al. [16] concerned smoking status for subgroup analysis and the results were in line with the present one. The precise mechanisms are not fully understood. A recent study has shown that MDM2 may act as an oncogene or tumor suppressor according to particular context [52]. MDM2 may bind P53 and promote P53 degradation at baseline but stimulate P53 translation under stress [53]. Therefore, because tobacco smoking may lead to severe DNA damage [54], two probable events may be noticeable. On the one hand, under this cellular stress, MDM2 may increase P53 mRNA via ATM pathway and result in elevated P53 protein that can inhibit tumorigenesis of lung tissues [55]. On the other hand, severe DNA damage may directly trigger apoptosis in lung cells via the P53-independent pathways [56]. Consequently, the possibility of lung carcinogenesis might be reduced. This might help determine the possible reasons why gene-smoking interaction analyses showed that smoking lowered the risk.

When the data were stratified according to ethnicity, no marked increased lung cancer risk was observed among Asians, Caucasians or Africans, inconsistent with the two previous meta-analyses [16], [17]. The results in the study by Bai et al. [16] regarding ethnicity were the same as the data by Gui et al. [17] and showed an increased lung cancer risk among Asians. Notably, Bai et al. [16] only reported the results under the recessive model and thus concluded that no significant elevated risk was found in Asians. However, in other genetic models such as dominant model, evident increased risk could be found among Asians. Compared with these two meta-analyses [16], [17], the present study failed to indicate an excess lung cancer risk among Asians. The data may be more convincible due to the much larger number of the included studies. Nevertheless, considering the ethnicity-specific chromosomal aberrations [57] and epidemiological variation [58] of lung cancer in the world, more investigations with large sample sizes are required for clarification of the potential differences among different races.

In subgroup analysis according to gender, no increased lung cancer risk was presented in the male group. However, the recessive model showed increased lung cancer susceptibility among female individuals carrying GG alleles. The underlying mechanisms are still unclear. Evidence suggests that estrogen receptors have been widely detected in lung cancer cells, indicating that sex steroid may play a critical role in the pathogenesis of lung neoplastic diseases [59], [60]. Besides, MDM2 may act as a strong contributor via the P53-independent pathway during the process of estrogen-induced cell proliferation [61]. MDM2 can induce expression of the p65 subunit of NF-κB, which is an anti-apoptotic factor expressed in neoplastic cells [62]. In addition, SNP309 of MDM2 increases the binding affinity for Sp1, a coactivator of receptors for multiple hormones including estrogen. It could potentially affect the hormone-dependent regulation of MDM2 transcription and result in further elevation of the MDM2 protein levels [63], [64]. Thus, MDM2 T309G genetic variation might accelerate carcinogenesis of lung tissues in a gender-specific manner [65]. This may also be one reason why women are potentially more vulnerable to lung cancer development [66], [67]. However, the results should be interpreted with care because increased lung cancer risk was not shown in the additive and dominant models. Therefore, further studies concerning stratification for gender could increase power for the association estimation.

In the subgroups concerning histological type, no significant associations were observed in either the adenocarcinoma or the squamous cell carcinoma group. The results were in line with the overall data. We tried to extract information about other types of lung cancer. However, only sufficient data regarding adenocarcinoma and squamous cell carcinoma are available in the primary literature. Considering that possible differences may exist between these two most common subtypes of lung carcinoma due to the differences in genetic changes during tumorigenesis [68], a number of large sample studies concerning the histological types were needed.

In the present study, between-study heterogeneities for overall data were observed in the three genetic models, and thus a random-effect model was used. However, we found that the heterogeneities were removed or reduced in the subgroup analyses, suggesting that the heterogeneities may be multifactorial such as selection of controls, race variation, gender, and prevalence of lifestyle factors.

Publication biases were assessed via funnel plots and their symmetries were further evaluated by Egger’s linear regression tests. The data suggest that no evident biases were observed in the three genetic models, indicating the credibility and robustness of the results.

Whether MDM2 SNP309 polymorphism has a correlation with the prognosis of cancers remains controversial. Evidence indicates that MDM2 SNP309 genetic variation might confer poor outcomes in colorectal cancer and chronic lymphocytic leukemia [69], [70]. However, a study regarding prostate carcinoma failed to reveal such association [71]. In the present meta-analysis, only one included study conducted by Chua et al. [30] concerned the prognosis of lung carcinoma and showed no effect of MDM2 SNP309 polymorphism on the overall survival. Thus, the association between MDM2 SNP309 polymorphism and lung cancer outcomes has not been evaluated because of the insufficient information provided by the included studies. Future investigations on the issue are required.

Several limitations might be included in this study. First, in this meta-analysis, the subgroup analysis concerned only Caucasians, Asians and African. Data regarding African could be extracted from only one study. Thus, data regarding other ethnicity are desired. Second, subgroup analyses regarding age have not been conducted in the present study because the criteria for age division were different in the primary literature. Third, hospital-based controls were used in some included studies and hence, non-differential misclassification bias might exist. Nevertheless, subgroup analysis regarding source of controls was carried out and no evident influence on the results was found. Additionally, gene-gene and gene-environment interactions should also be considered in future studies. However, the sensitivity analysis and publication biases analysis suggest the stability and credibility of the present meta-analysis.

In summary, previous meta-analyses indicated a possible association of MDM2 T309G polymorphism with lung cancer risk among Asians and never-smokers. In the present updated meta-analysis, the data confirmed the relationship between MDM2 T309G genetic variation and lung cancer risk among individuals who have no smoking history. However, the data failed to show a significant increased lung carcinoma risk among Asians. More interestingly, subgroup analysis regarding histological types, source of controls and sex were performed and the data suggest that homozygous GG alleles might elevate lung cancer risk among females. Further investigations are needed to confirm the conclusions.
